# Development and evaluation of soy lecithin-derived nanoliposomes as a plant-based alternative to egg-yolk extender for Ongole-grade bull semen cryopreservation

**DOI:** 10.14202/vetworld.2025.3433-3446

**Published:** 2025-11-23

**Authors:** Muhammad Gunawan, Ni Wayan Kurniani Karja, Mohamad Agus Setiadi, Ekayanti Mulyawati Kaiin, Syahruddin Said, Raden Iis Arifiantini, Hikmayani Iskandar

**Affiliations:** 1 Graduate School of Reproductive Biology, School of Veterinary Medicine and Biomedical Sciences, IPB University, Bogor 16680, West Java, Indonesia; 2 Division of Reproduction and Obstetrics, School of Veterinary Medicine and Biomedical Sciences, IPB University, Bogor 16680, West Java, Indonesia; 3 Research Center for Applied Zoology, National Research and Innovation Agency (BRIN), Jalan Raya Jakarta-Bogor KM 46, Cibinong, Bogor 16911, West Java, Indonesia

**Keywords:** computer-assisted semen analysis, cryopreservation, lecithin, nanoliposome, Ongole-grade bull, semen extender, sperm integrity

## Abstract

**Background and Aim:**

Conventional egg-yolk and milk-based extenders are widely used for semen cryopreservation but pose biosafety concerns and compositional variability that compromise standardization. Liposome technology offers a biosecure, uniform alternative. This study aimed to develop soy lecithin-derived nanoliposomes (NLs) using an ultrasonic-based process and to evaluate their efficacy as a Tris-based extender for Ongole-grade bull semen cryopreservation.

**Materials and Methods:**

Soy lecithin NLs were prepared through probe ultrasonication (15–45 min) and ultracentrifugation, followed by physicochemical characterization using particle size analysis, scanning electron microscopy (SEM), and high-resolution transmission electron microscopy. Tris-NL (TNL) extenders were formulated at concentrations of 5–25 mg/mL and compared with a Tris–egg-yolk (TEY, 20%) control. Fresh semen from five Ongole-grade bulls was evaluated for motility, viability, and morphology before and after freezing. Kinematic parameters were assessed through computer-assisted semen analysis, plasma membrane integrity by hypoosmotic swelling test, acrosome integrity using fluorescein isothiocyanate-conjugated peanut agglutinin/propidium iodide staining, and DNA fragmentation by Halomax-sperm chromatin dispersion assay.

**Results:**

Optimized sonication (45 min) produced stable NLs (mean diameter 76 nm, zeta potential −43.2 mV) with uniform spherical morphology. Among the tested formulations, TNL 5–10 mg/mL showed significantly higher (p < 0.05) post-equilibration motility (up to 98%), progressive motility, and kinematic parameters (velocity curved line, velocity average path, and velocity straight line) than TEY. Post-thaw evaluations demonstrated improved sperm viability (≈66%), reduced abnormalities (<7%), enhanced plasma-membrane and acrosomal integrity, and lower DNA fragmentation (~1.2%) in the 5–10 mg/mL groups. SEM confirmed smoother sperm surfaces with minimal cryo-damage compared with TEY.

**Conclusion:**

Soy lecithin-derived NLs at 5–10 mg/mL serve as an effective and biosecure substitute for egg yolk in Tris extenders, enhancing motility, viability, and structural integrity of Ongole-grade bull spermatozoa. This locally developed, plant-based nanotechnology supports biosafety, import substitution, and sustainability of artificial insemination programs in Indonesia.

## INTRODUCTION

Semen cryopreservation is a critical biotechnological approach for the long-term preservation of spermatozoa in liquid nitrogen (LN_2_) at −196°C [1]. However, the freezing and thawing cycle frequently induces cryoinjury to the plasma membrane, cytoskeleton, mitochondria, and nuclear DNA due to oxidative stress and excessive generation of reactive oxygen species [2]. Such cellular damage compromises genome stability, fertilization potential, and early embryonic development [3, 4]. The efficiency of cryopreservation largely depends on the physicochemical composition of the semen extender, including the type and concentration of cryoprotectant, cooling and thawing rates, and key properties, such as pH and osmolality [5]. An ideal extender should maintain isotonic balance, minimize cold and freeze shock, and exhibit antioxidant capacity to protect sperm cells throughout the freezing process [6].

Lecithin-based semen extenders are known for their anti-cold-shock properties and have been developed from various sources, including egg yolk, milk, soy, and synthetic liposomes. These components form a protective layer around sperm cells, stabilizing the plasma membrane and preventing intracellular ice crystal formation during freezing [7, 8]. Despite their effectiveness, extenders derived from egg yolk and milk present notable disadvantages: they are animal-origin materials that carry microbiological risks, such as potential pathogen transmission [9], and exhibit biochemical variability that complicates standardization [7]. Furthermore, these substances may interfere with sperm metabolism and respiration, reduce motility, and affect semen analysis accuracy [10].

To address these limitations, liposomes have been introduced as promising alternatives to conventional animal-derived extenders. Liposomes are advantageous due to their biocompatibility, biodegradability, and non-toxic nature. Their nanoscale size and amphiphilic properties allow spontaneous formation of bilayer vesicles capable of encapsulating and delivering bioactive compounds [11]. Liposome-based cryoprotectants derived from pure phospholipids have been successfully applied in cattle (*Bos taurus*) [12, 13], buffalo (*Bubalus bubalis*) [13], and sheep (*Ovis aries*) [14]. These artificial liposomes are typically produced through sonication, which disrupts lipid aggregates to yield uniform nanovesicles smaller than 100 nm [15]. The adoption of nanotechnology in reproductive biology leverages these distinctive physicochemical features to enhance sperm preservation [16].

Although liposome-based commercial extenders exist, most are still imported into Indonesia. Given that the country operates 21 artificial insemination (AI) centers producing frozen semen [17], variations in extender composition have resulted in inconsistent semen quality. Therefore, the development of standardized, plant-based lecithin extenders, such as soy-derived nanoliposomes (NL), represents a crucial step toward improving semen stability, biosafety, and long-term storage in Indonesian breeding programs.

Despite the well-documented benefits of liposome-based extenders for semen cryopreservation, significant scientific and practical gaps remain. Most existing liposome formulations rely on purified or synthetic phospholipids, which are imported at a high cost, thereby limiting accessibility for local AI centers in developing countries. Moreover, studies in cattle have primarily focused on *B. tauru*s breeds under temperate conditions, leaving limited data on the physicochemical stability and cryoprotective performance of NLs in *Bos indicus-*derived breeds such as the Ongole grade, which differ in sperm membrane lipid composition and cold-shock susceptibility. In Indonesia, the lack of locally produced lecithin-based extenders contributes to variability in semen quality among AI centers, as each center uses different extender formulations and imported components. Furthermore, while several reports have evaluated post-thaw motility and viability, few have integrated advanced assessments of membrane integrity, acrosomal status, DNA fragmentation, and kinematic parameters using computer-assisted semen analysis (CASA). These limitations hinder the establishment of a standardized, biosafe, and cost-effective cryopreservation protocol suited to national breeding programs.

Therefore, this study aimed to develop, characterize, and evaluate soy lecithin-derived NLs as a plant-based alternative to conventional egg-yolk extenders for Ongole-grade bull semen cryopreservation. Specifically, the objectives were to:

1. Produce and characterize NLs using an ultrasonic-assisted method and determine their physicochemical properties (particle size, zeta potential, and morphology).
2. Formulate Tris-based NL (TNL) extenders at different concentrations (5–25 mg/mL) and compare their performance with a conventional Tris–egg-yolk (TEY) extender.
3. Assess sperm quality parameters before and after cryopreservation, including motility, kinematics (velocity curved line [VCL], velocity average path [VAP], velocity straight line [VSL], beat cross frequency [BCF], and amplitude of lateral head displacement [ALH]), viability, plasma membrane integrity (PMI), acrosome integrity, and DNA fragmentation, using advanced CASA and staining techniques.
4. Identify the optimal TNL concentration that maintains post-thaw sperm quality and structural integrity comparable to or superior to the egg-yolk-based extender.

By integrating nanotechnology and reproductive biotechnology, this study seeks to establish a standardized, biosecure, and sustainable extender suitable for local AI programs, thereby reducing dependence on imported reagents and supporting Indonesia’s goals for genetic resource conservation, livestock productivity, and compliance with Sustainable Development Goal (SDG) 2 (Zero Hunger) and SDG 12 (Responsible Consumption and Production).

## MATERIALS AND METHODS

### Ethical approval

All experimental procedures involving animals were reviewed and approved by the Animal Ethics Committee of the National Research and Innovation Agency (BRIN), Indonesia (Approval No. 166/KE.02/SK/07/2024). The study was conducted in accordance with the ethical standards established by BRIN’s institutional animal welfare guidelines, the Animal Research: Reporting of *In Vivo* Experiments (ARRIVE) 2.0 framework, and the World Organization for Animal Health (WOAH, 2019) recommendations for the ethical use of animals in research.

No invasive procedures beyond routine semen collection using an artificial vagina were performed. All bulls were maintained under standard animal husbandry conditions with ad libitum access to feed and water, and efforts were made to minimize discomfort, stress, and handling time during sampling. The number of animals used was limited to the minimum required to achieve statistical validity. This research complied with national laws and institutional policies for animal experimentation and biosafety, ensuring that animal welfare was upheld throughout the study.

### Study period and location

The study was conducted from January to August 2024. The semen collection, evaluation, and preparation of the Tris-based semen extender were carried out at the Reproduction Laboratory, Genomic Building, BRIN, Bogor, Indonesia. The assessment of sperm quality was performed at the Reproduction Laboratory, School of Veterinary Medicine and Biomedical Sciences, IPB University, Bogor, Indonesia.

### Animal management

Fresh semen samples were obtained from five Ongole-grade bulls aged between 8 and 10 years. The collected semen was transported to the laboratory for both macroscopic and microscopic analyses. Ongole-grade bulls were managed in accordance with BRIN’s standard operating procedures. Each bull was housed individually in a 2 × 3 m pen equipped with feed and water containers. The feeding regimen consisted of 10% fresh forage and 1.5% of concentrate per body weight twice a day, once in the morning and once in the evening, and water was provided *ad libitum*.

### Preparation of NL

#### Liposome preparation through ultrasonic probe

NLs were prepared using soy lecithin (phosphatidylcholine, ≥99%, Sigma-Aldrich) as the primary lipid component. A stock dispersion was prepared by dissolving 10 g of soy lecithin in 100 mL of sterile deionized water, resulting in a concentration of 100 mg/mL (w/v). The mixture was pre-homogenized using an Ultra-Turrax homogenizer (IKA T25, Germany) at 5,590 × *g* for 5 min to obtain a uniform emulsion. Subsequently, the pre-homogenized dispersion was subjected to sonication using a probe-type ultrasonic processor (Bioblock Scientific, France) operating at 750 W and 20 kHz frequency [18]. Sonication was performed in pulse mode (5 s on, 1 s off) at 100% amplitude while maintaining the temperature on ice between 18°C and 40°C to prevent thermal degradation. Three sonication durations (15, 30, and 45 min) were tested to optimize particle size. The suspensions were centrifuged at 8,000 × *g* for 60 min at 4°C using a refrigerated ultracentrifuge (Thermo Scientific Sorvall, USA) after sonication. The resulting supernatant containing the NL was collected and filtered through a 0.22 μm syringe filter (Millipore, USA) to achieve sterility. The NL stock solution was stored at 4°C and remained stable for up to 12 months without visible precipitation or change in zeta potential. However, to ensure reproducibility and consistency, a fresh working batch of NL-based extender was prepared for each semen collection.

#### Particle size and morphological analysis

The mean particle size and zeta potential of the NL were determined using a Particle Size Analyzer and Zeta Potential Analyzer (Horiba SZ-100, Horiba Scientific, Japan). For morphological characterization, the samples were examined under a scanning electron microscope (SEM) (Prisma E, Thermo Scientific, USA) and a high-resolution transmission electron microscope (HR-TEM) (Talos F200C, Thermo Scientific).

### Formulation of Tris-based extenders

#### Extender composition

The Tris-based semen extender formulations are shown in Table 1. The control group contained Tris buffer with 20% (v/v) egg yolk, whereas five treatment groups contained NL at concentrations of 5, 10, 15, 20, and 25 mg/mL, respectively. Each formulation contained 7% (v/v) glycerol and was adjusted to a final volume of 10 mL. All ingredients were dissolved in sterile deionized water under continuous stirring. The pH of the extender was measured using a pH meter (Eutech Instruments, Singapore), and osmolality was verified using an osmometer (Osmomat 3000, Gonotec, Germany).

**Table 1:** Composition of tris-based semen extenders.

Extender type	Tris buffer (mL)	Nano-liposome (mL)	Egg yolk (mL)	Glycerol 7% (mL)	Total volume (mL)
Tris + egg yolk 20% (control)	7.3	-	2.0	0.7	10.0
Tris + NL (5 mg/mL)	8.8	0.5	-	0.7	10.0
Tris + NL (10 mg/mL)	8.3	1.0	-	0.7	10.0
Tris + NL (15 mg/mL)	7.8	1.5	-	0.7	10.0
Tris + NL (20 mg/mL)	7.3	2.0	-	0.7	10.0
Tris + NL (25 mg/mL)	6.8	2.5	-	0.7	10.0

Nanoliposomes were prepared at a stock concentration of 0.1 g/mL (100 mg/mL) by dispersing 10 g of liposomes in 100 mL of sterile deionized water. Each treatment was adjusted to achieve the desired NL concentrations by calculating the appropriate dilution from the stock dispersion (e.g., 0.5 mL of stock provides 50 mg NL in 10 mL = 5 mg/mL). Egg yolk was omitted in all NL-containing treatments to evaluate its potential as a plant-based alternative. NL = Nano-liposome.

### Cryoprotectant and antibiotic supplementation

Glycerol (≥99.5%, analytical grade; Merck, Germany) was added as a cryoprotectant at a final concentration of 7% (v/v) in all extender formulations. To prevent microbial contamination, a combination of antibiotics (Sigma-Aldrich) was incorporated, consisting of gentamicin (500 μg/mL), tylosin (100 μg/mL), lincomycin (300 μg/mL), and spectinomycin (600 μg/mL). All antibiotics were thoroughly mixed into the buffer solution before the addition of egg yolk or NLs to ensure complete homogeneity within the extender.

### Measurement of pH and osmolality

The pH of each extender was measured using a calibrated digital pH meter (Eutech Instruments), and osmolality was determined by a freezing-point osmometer (Osmomat 3000, Gonotec). The pH and osmolality were maintained within the ranges of 6.8–7.0 and 280–300 mOsm/kg, respectively, to ensure optimal sperm viability and membrane integrity during cryopreservation.

### Fresh semen collection and analysis

#### Collection procedure

Fresh semen from Ongole-grade bulls was collected twice weekly in the morning using an artificial vagina. For each bull, three ejaculates were obtained on different collection days and subsequently pooled to minimize individual variability. The pooled semen from each bull was then analyzed in triplicate for all macroscopic and microscopic parameters following the procedures described by Arifiantini [19]. Immediately after collection, the semen samples were transported to the laboratory for evaluation under controlled temperature conditions.

#### Macroscopic and microscopic evaluation

Macroscopic analysis encompassed the measurement of semen volume, color, consistency, and acidity (pH). Microscopic evaluation included the assessment of sperm motility, kinematic parameters, concentration, morphology, and viability. Sperm motility and kinematics were analyzed using a CASA system (SpermVision, Minitüb, Germany) following standard protocols [20, 21]. Sperm concentration was determined using a spectrophotometer (SDM 6, Minitüb, Germany), while morphology and viability were evaluated through differential staining under a light microscope at 400× magnification. Only ejaculates that met the predefined quality criteria, with a minimum sperm concentration of 1,000 × 10^6^ cells/mL and total motility of ≥70%, were included in this study.

### Semen dilution, packaging, and freezing

#### Dilution and treatment groups

The pooled semen samples were divided into six treatment groups, consisting of one control group diluted with TEY extender and five experimental groups supplemented with TNL extenders containing 5, 10, 15, 20, and 25 mg/mL (w/v) of NLs. Semen dilution was performed in a single-step process at room temperature (22°C–25°C) to achieve a final concentration of 25 × 10^6^ spermatozoa per 0.25 mL straw. All mixing procedures were conducted gently and uniformly to minimize mechanical stress and maintain sperm integrity. After dilution, the extended semen samples were placed in a water bath at 35°C and gradually cooled to 4°C over 60 min, corresponding to a cooling rate of approximately 0.51°C/min. The samples were maintained at 5°C for an additional 180 min to complete equilibration, resulting in a total equilibration time of 4 h. Subsequently, a pre-freezing step was carried out by reducing the temperature from 4°C to −120°C over 15 min (equivalent to a cooling rate of approximately 8.3°C/min) before immersion in LN_2_ (−196°C) for storage.

#### Packaging and equilibration

The diluted semen samples were packaged into 0.25 mL French straws using an automated filling and sealing machine (MPP Quatro, Minitüb, Germany). Each straw was labeled according to the treatment group using a laser printing machine (Jet 2, Minitüb). The straws were then horizontally arranged on a freezing rack and subjected to an equilibration period of 4 h at 4°C to allow proper glycerol permeation and membrane stabilization before freezing.

### Freezing and long-term storage

After equilibration at 4°C, the semen straws were frozen using the LN_2_ vapor-phase method. The straws were positioned approximately 6 cm above the LN_2_ surface in a cryobox and exposed to nitrogen vapor at −120°C for 15 min, corresponding to an estimated cooling rate of 20°C–25°C/min from room temperature (22°C–25°C) to 4°C before freezing. Controlled vapor-phase freezing was selected to avoid thermal shock and minimize intracellular ice formation. Following vapor exposure, the straws were plunged directly into LN_2_ (−196°C) for long-term storage. All frozen samples were maintained in LN_2_ storage tanks under continuous monitoring and were evaluated after a standard storage period of 7 days to assess post-thaw sperm quality parameters.

### Post-thaw evaluation of sperm quality

#### Thawing procedure

Frozen semen straws were thawed in a water bath at 37°C for 30 s. Immediately after thawing, the straws were gently wiped to remove surface moisture, and both sealed ends (manufacturer and laboratory seals) were carefully cut to expel the semen into a 1.5 mL microcentrifuge tube (CentriStar microtube, Corning Inc., USA). The semen samples were then incubated at 37°C to preserve physiological temperature during subsequent microscopic and biochemical evaluations.

#### CASA analysis of motility and kinematics

Sperm motility was performed using a CASA (SpermVision 3.7, Minitüb, Tiefenbach). Sperm motility was assessed by placing a 5 μL aliquot of semen onto a pre-warmed (37°C) glass slide, which was then covered with a coverslip. Video recordings were captured at 30 frames/s for 1 s under a phase-contrast microscope (200× magnification). The analysis was conducted at 37°C using the following settings: minimum contrast of 60, minimum cell size of 55 pixels, and a threshold of five tracks per field. The motility parameters evaluated included total motility (%), progressive motility (PM) (%), (VCL, μm/s), (VAP, μm/s), (VSL, μm/s), (BCF, Hz), and (ALH, μm). A minimum of ten microscopic fields were analyzed per sample, and approximately 1,000 spermatozoa were assessed to obtain mean values for each parameter [20, 21].

#### Sperm viability and morphology

Sperm viability and morphological abnormalities were evaluated using the eosin-nigrosin staining technique following standard procedures [22, 23]. Sperm viability was characterized by the absence of dye absorption by live sperm, while dead sperm exhibit dye absorption. Morphological evaluation was performed under light microscopy to identify abnormalities in the heads, acrosome, midpiece, and tail regions, including detached heads, nuclear vacuoles, abnormal acrosomes, proximal cytoplasmic droplets, midpieces, and tails [24].

#### PMI

PMI was evaluated using the hypo-osmotic swelling (HOS) test as described by Iskandar *et al*. [22]. Briefly, 10 μL of semen was added to 1 mL of HOS solution (1:100), gently mixed, and incubated at 37°C for 30–45 min. After incubation, a drop of the mixture was placed on a glass slide, covered with a coverslip, and examined under a light microscope at 400× magnification. Spermatozoa displaying coiled or swollen tails were classified as having intact plasma membranes, whereas those with straight tails were classified as membrane-damaged.

#### Acrosome integrity (fluorescein isothiocyanate-conjugated peanut agglutinin/propidium iodide [FITC-PNA/PI] staining)

Acrosomal integrity was evaluated using (FITC-PNA; Sigma-Aldrich, St. Louis, MO, USA) according to the manufacturer’s protocol described by Hasbi *et al*. [25]. Semen smears were prepared on glass slides, fixed in 96% ethanol for 10 min at room temperature (22°C–25°C), and air-dried. Subsequently, 30 μL of FITC-PNA solution (100 μg/mL) was applied to each slide and incubated at 37°C for 30 min. After incubation, 5 μL of PI (1 μg/μL, Sigma-Aldrich) was added for nuclear counterstaining and incubated for 5 min. The slides were then washed 3 times with phosphate-buffered saline to remove unbound reagents and mounted with a coverslip. Fluorescence microscopy was performed using an ImagerZ2 Zeiss microscope (Oberkochen, Germany) equipped with a 490/530 nm excitation/emission filter and a 380/420 nm barrier filter. A total of 200 sperm cells per slide were examined. Spermatozoa exhibiting green fluorescence localized to the acrosomal region were classified as having intact acrosomes, whereas those showing red fluorescence due to PI uptake were considered damaged.

#### DNA fragmentation (Halomax-sperm chromatin dispersion [SCD] test)

DNA fragmentation in frozen semen was evaluated using the modified SCD method with the Halomax kit (Halotech DNA, Madrid, Spain) following the procedure described by Prabowo *et al*. [26] and Asir *et al*. [27]. A total of 200 sperm per slide were examined under bright-field microscopy. Sperm displaying a distinct halo of dispersed DNA loops around the sperm head were classified as having fragmented DNA, whereas those lacking a halo were considered to have intact DNA.

### Preparation for SEM

Samples were first cleaned by immersion in cacodylate buffer (0.2 M, pH 8.4) for approximately 2 h with gentle agitation using an ultrasonic cleaner. Prefixation was carried out in 2.5% glutaraldehyde prepared in cacodylate buffer for 1–2 days at 4°C, followed by fixation in 2% tannic acid for 6 h to several days. The specimens were rinsed 4 times with cacodylate buffer and then subjected to graded ethanol dehydration (50%, 70%, 85%, 95%, and absolute ethanol) at 22°C–25°C. Subsequently, the samples were immersed in tert-butanol for 10 min, frozen, and freeze-dried until completely dry. Dried specimens were mounted on aluminum stubs using a conductive adhesive and sputter-coated with gold using an ion coater before SEM observation. All preparations were performed at 4°C unless otherwise specified.

### HR-TEM analysis

HR-TEM analysis was performed using a Talos F200C instrument (Thermo Fisher Scientific, USA) operated at 200 kV accelerating voltage. The samples were prepared as thin lamellae with a thickness of <100 nm to allow electron beam transmission. HR-TEM imaging was conducted to observe the ultrastructural morphology and crystallinity of the specimens at magnifications up to 121,000×. The instrument was equipped with an Energy Dispersive X-ray detector for elemental composition analysis and a High-Angle Annular Dark Field mode for high-contrast imaging. The system enabled simultaneous STEM and HR-TEM acquisition, providing detailed information on particle size, lattice fringes, and atomic arrangement. All analyses were performed under vacuum conditions, and data were digitally captured for subsequent structural interpretation.

### Statistical analysis

All quantitative data were analyzed using one-way analysis of variance to determine differences among treatment groups. The results obtained were tabulated and presented in mean ± standard deviation. When significant differences were detected (p < 0.05), Duncan’s multiple range *post hoc* test was applied. Statistical analyses were performed using the Statistical Package for the Social Sciences software version 25.0 (IBM Corp., Armonk, NY, USA) and Microsoft Excel 2019 (Microsoft Office, Washington, USA).

## RESULTS

### Characteristics of the NL

**Figure 1:**
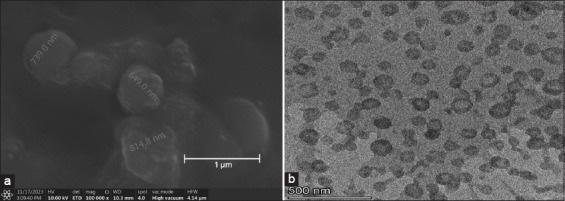
(a) The morphology of nanoliposomes was examined using scanning electron microscopy at a magnification of 100,000×. (b) The morphology of nanoliposomes examined using high-resolution transmission electron microscopy at a magnification of 36,000×.

Table 2 summarizes the dimensions and stability of the NLs. The particle size analysis (PSA) of NL, after 45 min of sonication and 60 min of centrifugation at 5,590 × *g*, revealed an average particle size of 76 nm, placing it within the nanoparticle size range of <100 nm. The morphology of NL observed through SEM showed a spherical shape (Figure 1a). The morphology of the NLs was also examined using high-resolution TEM (Figure 1b).

**Table 2:** Particle size and zeta potential of NL based on particle size analysis and ZP.

Treatment	Particle size (nm)	ZP (mV)
NL-15	117.0^c^	−39.2^c^
NL-30	109.3^b^	−42.6^b^
NL-45	76.0^a^	−43.2^a^

Different letters within the same variable indicate a significant difference (p < 0.05). ZP = Zeta potential, NL = Nano-liposome, NL-15 = Nano liposomes sonication 15 min, NL-30 = Nano liposomes sonication 30 min, and NL-45 = Nano liposomes sonication 45 min.

### Characteristics of the extender

Table 3 presents the acidity (pH) and osmolality of the extender formulations used in this study. The results indicate that the pH values across all extender formulations remained within the normal range (6.5–7.5) and showed no significant differences. Fluctuations in pH, whether toward more acidic or alkaline levels, could negatively impact sperm cell membrane integrity and extender properties.

**Table 3:** The acidity and osmolality values of the extender formulation.

Tris-base extender	pH	Osmolality (mOsmol/kg)
Tris egg yolk 20% (TEY)	6.86 ± 0.01	311.50 ± 1.50^a^
Tris nanoliposome (TNL) 5 mg/mL	6.80 ± 0.02	310.00 ± 2.50^a^
TNL 10 mg/mL	6.82 ± 0.05	302.00 ± 1.00^b^
TNL 15 mg/mL	6.83 ± 0.04	300.75 ± 1.75^b^
TNL 20 mg/mL	6.82 ± 0.03	295.25 ± 3.75^c^
TNL 25 mg/mL	6.83 ± 0.06	291.50 ± 1.00^c^
TEY 20% + 7% glycerol	6.87 ± 0.01	1707.75 ± 5.88^a^
TNL 5 mg/mL + 7% glycerol	6.81 ± 0.02	1403.50 ± 9.00^b^
TNL 10 mg/mL + 7% glycerol	6.83 ± 0.05	1411.25 ± 8.38^b^
TNL 15 mg/mL + 7% glycerol	6.84 ± 0.04	1349.75 ± 4.88^c^
TNL 20 mg/mL + 7% glycerol	6.83 ± 0.04	1353.50 ± 6.50^c^
TNL 25 mg/mL + 7% glycerol	6.85 ± 0.06	1271.50 ± 3.00^d^

Different letters in the same variable indicate a significant difference (p < 0.05).

In terms of osmolality, the extender group without glycerol exhibited significantly higher values (p < 0.05) in both TEY and TNL at 5 mg/mL. The osmolality in this group ranged from 291.50 mOsmol/kg to 311.50 mOsmol/kg, which falls within the normal range of 280–320 mOsmol/kg. In addition, the frozen semen extender group containing 7% glycerol showed a significant difference in osmolality values (p < 0.05). Compared with the TNL formulation, the TEY formulation exhibited a higher osmolality. The addition of 7% glycerol to all extender formulations further increased osmolality, ranging from 1271.50 mOsmol/kg to 1707.75 mOsmol/kg.

### Quality of fresh semen

Table 4 shows that the fresh bull semen used in this study meets the quality standards outlined in SNI Frozen Semen from Cattle No. 4869.1-2021. The addition of NL to the Tris extender, replacing egg yolk, did not show a significant difference (p < 0.05) in the parameters of motility, progressiveness, viability, abnormality, and intact sperm membrane after freezing.

**Table 4:** Macroscopic and microscopic quality of fresh semen.

Parameters	Values
Volume (mL)	5.3 ± 0.24
Consistency	Moderate
Color	Cream
pH	6.97 ± 0.04
Mass movement	+++
Sperm concentration (×10^6^/mL)	1.526 ± 0.11
Total motility (%)	96.59 ± 1.61
Progressive motility (%)	92.20 ± 2.55
Viability (%)	86.70 ± 1.44
Abnormality (%)	6.77 ± 0.81
Plasma membrane integrity (%)	88.73 ± 1.32

The kinematic values (VCL, VAP, VSL, BCF, and ALH) were significantly higher (p < 0.05) in the 1% TNL extender than in the TEY control. Sperm motility after thawing in all treatments was >40%, indicating that both TEY and TNL extenders could maintain sperm quality during freezing.

### Quality of bull semen in the TNL extender at the equilibration stage

The equilibration stage is essential for sperm adaptation before freezing, and the semen quality after this process is outlined in Table 5. Semen treated with the TNL extender at 10 mg/mL showed the highest post-equilibration quality, with significantly better results (p < 0.05) across all parameters compared to the TEY extender. Specifically, the 10 mg/mL TNL extender led to significantly higher total motility and PM values (p < 0.05) than the control and other concentrations.

**Table 5:** Bull semen quality in TNL extender after equilibration.

Variable	TEY 20% (control)	TNL 5 mg/mL	TNL 10 mg/mL	TNL 15 mg/mL	TNL 20 mg/mL	TNL 25 mg/mL
Total motility (%)	86.85 ± 2.21^c^	94.07 ± 1.38^b^	98.41 ± 0.56^a^	93.00 ± 2.35^b^	90.87 ± 3.00^b^	85.57 ± 2.25^c^
Prog motility (%)	80.12 ± 3.00^c^	86.96 ± 1.80^b^	94.10 ± 1.08^a^	87.39 ± 2.02^b^	84.83 ± 3.34^b^	74.97 ± 3.50^d^
VCL (mm/s)	136.09 ± 11.20^c^	169.07 ± 17.39^b^	204.43 ± 15.09^a^	159.24 ± 9.11^b^	170.13 ± 10.28^b^	152.49 ± 10.32^bc^
VAP (mm/s)	88.17 ± 6.03^c^	100.81 ± 8.16^b^	120.31 ± 5.24^a^	100.06 ± 4.73^b^	104.91 ± 6.00^b^	95.78 ± 5.17^bc^
VSL (mm/s)	53.16 ± 2.80^d^	54.92 ± 3.73^cd^	64.17 ± 1.93^a^	58.55 ± 3.73^bc^	60.09 ± 1.59^ab^	55.18 ± 2.00^bcd^
BCF (Hz)	26.41 ± 0.33^b^	26.62 ± 0.61^ab^	27.97 ± 0.70^a^	26.67 ± 1.58^ab^	27.14 ± 0.75^ab^	25.77 ± 0.38^b^
ALH	6.97 ± 0.20^c^	8.25 ± 0.43^ab^	8.73 ± 0.70^a^	7.60 ± 0.37^bc^	7.95 ± 0.47^ab^	7.45 ± 0.40^bc^
Viability (%)	82.94 ± 1.10^b^	87.64 ± 1.96^a^	88.83 ± 2.49^a^	89.97 ± 1.21^a^	88.81 ± 0.36^a^	90.66 ± 1.06^a^
Abnormality (%)	9.40 ± 0.55	7.94 ± 1.91	7.85 ± 1.55	7.22 ± 1.53	8.72 ± 1.19	8.81 ± 0.57
PMI (%)	86.32 ± 0.94^d^	87.08 ± 0.62^cd^	88.53 ± 0.78^ab^	89.47 ± 0.46^a^	89.15 ± 0.51^a^	87.69 ± 0.15^bc^

Different letters within the same variable indicate a significant difference (p < 0.05). TEY = Tris egg yolk, TNL = Tris nanoliposome, VCL = Velocity curved line, VAP = Velocity average path, VSL = Velocity straight line, BCF = Beat cross frequency, ALH = Amplitude of lateral head displacement, PMI = Plasma membrane integrity.

Sperm viability was also higher in this group than in the control, but did not differ from other concentrations. No significant differences in sperm abnormalities were found across all extenders. In addition, sperm PMI remained unchanged for TNL extender concentrations between 10 mg/mL and 25 mg/mL, showing no significant difference from the control. However, the 5 mg/mL concentration group exhibited a significant increase in membrane integrity (p < 0.05).

Furthermore, the kinematic results of sperm at 10 mg/mL concentration on the variables VCL, VAP, VSL, BCF, and ALH were higher (p < 0.05) than those of the control, indicating that the measurement parameters of semen hyperactivity values were correlated with the speed and distance of sperm movement during 4 h of storage at 5°C.

The addition of 7% (v/v) glycerol to the extender functions as a permeable cryoprotectant for semen cryopreservation, protecting sperm at temperatures below the freezing point. In this study, the combination of TNL extender and glycerol successfully protected sperm cells during the cooling stage before freezing.

### Quality of bull semen in the TNL extender after freezing

A good semen freezing extender must be able to protect sperm cells during both freezing and thawing. Table 6 presents the quality of frozen semen after thawing. As demonstrated in Table 6, NL in the Tris extender is a suitable replacement for egg yolk.

**Table 6:** The quality of frozen semen of Ongole-grade bulls in the TNL extender after thawing.

Variable	TEY 20% (control)	TNL 5 (mg/mL)	TNL 10 (mg/mL)	TNL 15 (mg/mL)	TNL (20 mg/mL)	TNL (25 mg/mL)
Total motility (%)	63.27 ± 7.60^ab^	68.93 ± 3.56^a^	68.22 ± 4.62^a^	66.00 ± 1.48^a^	61.57 ± 4.45^ab^	56.53 ± 2.15^b^
Prog motility (%)	54.64 ± 8.45^ab^	62.29 ± 3.47^a^	58.14 ± 4.67^ab^	56.92 ± 2.14^ab^	55.53 ± 4.77^ab^	50.44 ± 1.84^b^
VCL (μm/s)	99.15 ± 6.65^c^	139.44 ± 10.83^a^	137.07 ± 12.19^a^	117.08 ± 5.41^b^	138.45 ± 7.95^a^	130.22 ± 6.36^ab^
VAP (μm/s)	42.67 ± 3.71^c^	57.82 ± 5.10^a^	58.11 ± 6.02^a^	48.05 ± 2.02^bc^	53.81 ± 5.36^ab^	51.26 ± 5.68^abc^
VSL (μm/s)	33.31 ± 3.14^c^	41.22 ± 3.70^abc^	43.92 ± 5.07^a^	35.19 ± 1.74^bc^	41.64 ± 4.40^ab^	39.83 ± 5.56^abc^
BCF (Hz)	11.93 ± 0.59^ab^	12.23 ± 0.30^ab^	12.80 ± 0.53^a^	11.59 ± 0.49^b^	12.75 ± 0.53^a^	12.52 ± 0.63^ab^
ALH	2.52 ± 0.13^c^	3.35 ± 0.20^a^	3.21 ± 0.23^a^	2.86 ± 0.11^b^	3.32 ± 0.12^a^	3.13 ± 0.05^a^
Viability (%)	65.29 ± 3.59^ab^	65.08 ± 2.53^ab^	66.03 ± 0.98^a^	65.34 ± 0.91^ab^	59.59 ± 4.59^bc^	57.79 ± 5.50^c^
Abnormality (%)	9.01 ± 1.47^a^	8.20 ± 1.37^ab^	6.98 ± 0.68^b^	8.74 ± 0.50^ab^	8.30 ± 0.61^ab^	8.95 ± 1.07^ab^
PMI (%)	60.15 ± 2.87^ab^	61.39 ± 2.03^ab^	64.75 ± 0.52^a^	62.18 ± 3.59^a^	54.51 ± 1.48^c^	56.76 ± 2.66^bc^
Acrosome integrity	98.11 ± 0.42^ab^	98.69 ± 0.46^a^	98.09 ± 0.24^ab^	97.97 ± 0.51^ab^	97.27 ± 0.70^bc^	96.74 ± 0.65^c^
DNA fragmentation	1.09 ± 0.35^b^	1.90 ± 0.20^b^	1.25 ± 0.40^b^	1.18 ± 0.51^b^	1.50 ± 0.49^ab^	2.18 ± 0.70^a^

TEY = Tris egg yolk, TNL = Tris nanoliposome, VCL = Velocity curved line, VAP = Velocity average path, VSL = Velocity straight line, BCF = Beat cross frequency, ALH = Amplitude of lateral head displacement, PMI = Plasma membrane Integrity. Different letters in the same rows are significantly different (p < 0.05).

## DISCUSSION

### Mechanistic significance of NL incorporation in Tris-based extenders

The incorporation of NLs into Tris-based extenders represents a novel and mechanistically informed approach for improving the cryopreservation outcomes of bull semen. In this study, the zeta potential (−43.2 mV) confirmed the strong electrostatic stability of the vesicles, as values below −30 mV typically indicate excellent colloidal stability [28]. Lower ZP values have been linked to shorter shelf life [29]. In addition, HR-TEM confirms that NL appears as spherical nanovesicles with a single lipid bilayer, supporting previous findings by Stremersch *et al*. [15]. This spherical morphology and nanovesicle structure suggest that NL may play a role in protecting the plasma membrane, the outermost layer of sperm cells.

To the best of our knowledge, this is the first study to locally develop and validate an NL-based semen extender for use in AI centers in Indonesia, offering a strategic step toward import substitution and biosafety. This combination of particle size optimization, zeta potential characterization, and vesicle morphology analysis links the present work to the broader framework of nanoengineering for reproductive biotechnology, where engineered nanostructures act not only as carriers but also as biofunctional systems that interact dynamically with sperm membranes.

### Role of osmolality and pH stability in cryoprotection

High osmolality levels make frozen semen extenders hyperosmolar, which is beneficial for removing and replacing intracellular fluid with cryoprotectants. This process helps regulate osmotic pressure and prevents the formation of intra- and extra-cellular ice crystals that could damage the sperm membrane during freezing [30]. However, osmotic stress can also harm sperm organelles during thawing [31, 32]. Maintaining stable pH and osmolality levels in extenders is essential for preserving the integrity of the plasma membrane, which protects sperm organelles.

This membrane stability is crucial for successful fertilization [33]. Inappropriate osmotic gradients can lead to rapid water fluxes across the membrane, resulting in cellular shrinkage or swelling and compromising membrane function. Moreover, even transient osmotic imbalances during thawing may disrupt the sperm plasma membrane’s lipid organization, impairing its ability to support acrosomal exocytosis, capacitation, and oocyte interaction. Therefore, optimizing the composition of extenders to maintain osmotic and pH homeostasis is critical for minimizing cellular injury during the cryopreservation cycle.

### Quality parameters of fresh semen before freezing

It satisfies the minimum requirement of 70% motility and contains 20% abnormal sperm [34]. Macroscopic evaluation confirms that key characteristics, such as volume, consistency, color, odor, and pH, are within the normal range [19]. The viability, abnormality, and PMI values of the fresh semen used were within the ranges of >80% [35], >60% [33], and <20% [34], respectively. These results indicate that fresh semen is of adequate quality for processing into frozen semen.

Maintaining high initial semen quality is essential because spermatozoa are susceptible to damage during cryopreservation, which can reduce motility, membrane integrity, and fertility potential. The likelihood of producing viable post-thaw spermatozoa is significantly increased by ensuring optimal pre-freezing parameters. Semen that meets pre-freezing quality thresholds is more likely to withstand osmotic and thermal stresses encountered during dilution, equilibration, and freezing stages.

### Comparative analysis of TEY and TNL extenders

Based on the TEY extender analysis using CASA, egg yolk particles of the size of sperm cells affected the assessment of motile sperm and resulted in less accurate values [36]. Higher viscosity in the extender media with egg yolk resulted in lower VAP, VCL, and VSL values [37]. Post-thaw ultrastructural evaluation using SEM demonstrated that spermatozoa cryopreserved with TNL 5–10 mg/mL extender exhibited superior membrane preservation compared with those extended with TEY 20%.

**Figure 2:**
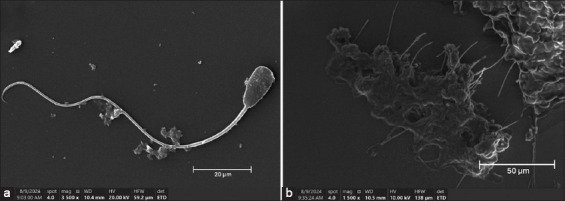
(a) Morphology of spermatozoa after post-thawing using tris–nano liposome 5 mg/mL extender, observed under scanning electron microscopy (SEM) at 3500× magnification. (b) Morphology of spermatozoa after post-thawing using Tris–egg yolk 20% extender, observed under SEM at 1500× magnification.

This was characterized by a smoother surface topology and a reduced incidence of structural abnormalities (Figure 2). These findings support the hypothesis that NLs provide a more homogenous and less obstructive medium for sperm motion analysis, thereby enhancing the accuracy of CASA. Furthermore, the lower viscosity and particle uniformity of NL-based extenders likely facilitate more consistent diffusion of cryoprotectants and reduce shear stress during handling and freezing. The enhanced membrane preservation observed under SEM suggests that NL-based extenders better maintain sperm membrane integrity during freeze–thaw cycles, likely due to their capacity to fuse with or stabilize lipid bilayers.

### Liposome–membrane interactions and cryostability mechanisms

The transition to NL in Tris-based extenders has been observed to enhance the bioavailability of functional materials compared with conventional lecithin [38]. NL interacts with the sperm plasma membrane, thereby reducing the damage caused by the freezing and thawing process [39]. The cooling and freezing process is characterized by a lipid-to-gel stage transition, which depends on the plasma membrane’s lipid composition.

The fusion of liposomes within the semen extender promotes the transfer of lipids and cholesterol, leading to the rearrangement of sperm membrane components and alterations in its physicochemical properties, thereby enhancing sperm cryostability [12, 40]. This study combines nanoengineering characterization techniques with reproductive biotechnology, offering mechanistic insights into liposome–sperm membrane interactions.

### Functional benefits of NL supplementation

The addition of organic nanoparticle materials, such as liposomes, to semen extenders reduces sperm cell death during cryopreservation. We propose that NLs may fuse with the sperm plasma membrane, thereby restoring lipid bilayer fluidity and reducing cryo-induced membrane phase transitions. Cryo-induced damage due to cold shock and osmotic stress during freezing and thawing, which compromises mitochondrial function and flagellar activity.

The ability of soy lecithin-derived NLs to bind to sperm membranes and their intrinsic antioxidant properties offer enhanced protection against structural and functional damage in bovine sperm [12, 13]. Furthermore, NLs, which function as extracellular vesicle-like structures, improve sperm motility, viability, mitochondrial activity, and membrane integrity following thawing [41].

### Preservation of acrosome and genomic integrity

Acrosome integrity assessment further demonstrates that TNL extenders can preserve acrosomal structures, as damage levels exceeding 50% significantly impair fertilizing ability [42]. A strong correlation between plasma membrane protection and acrosome preservation in semen treated with NL-based extenders has also been identified [43]. The development of cost-effective TNL formulations using locally sourced soy lecithin not only supports biosafety but also offers a sustainable alternative to imported commercial products [44]. In addition, NL extenders were associated with reduced sperm DNA fragmentation, suggesting their role in stabilizing the mitochondrial membrane and limiting oxidative damage to DNA chains, key factors for maintaining sperm genomic integrity [45, 46].

### Local innovation, sustainability, and broader applications

From a local innovation and sustainability standpoint, this study highlights the development of cost-effective NL extenders as an alternative to locally sourced soy lecithin. This approach strengthens Indonesia’s biotechnological independence and provides a scalable model for other developing countries seeking affordable, safe, and sustainable semen-preservation technologies. By reducing the reliance on imported cryoprotectants and animal-origin products, this study directly contributes to the national goals for food security and genetic resource conservation. Furthermore, the findings have translational potential across species. The demonstrated ability of TNL to maintain sperm membrane and acrosomal integrity suggests applicability to other domestic animals, such as buffalo, goats, and sheep, and potentially to wildlife species conservation where gamete cryopreservation is crucial for preserving genetic diversity. This broadens the relevance of the current findings beyond cattle, positioning TNL as a versatile cryoprotective platform.

## CONCLUSION

This study demonstrated that the incorporation of NLs derived from soy lecithin into Tris-based extenders offers a promising alternative to conventional egg yolk formulations for bull semen cryopreservation. The optimized NL formulation exhibited a mean particle size of 76 nm and a zeta potential of −43.2 mV, confirming high electrostatic stability and favorable colloidal dispersion. Morphological assessments using SEM and HR-TEM revealed spherical, unilamellar nanovesicles capable of interacting dynamically with sperm membranes. The extender formulations maintained stable pH (6.5–7.5) and osmolality (280–320 mOsmol/kg), which are essential for preserving sperm membrane integrity and preventing osmotic shock during freezing and thawing.

During equilibration and post-thaw evaluation, the TNL extender at 10 mg/mL achieved significantly higher total and PM, viability, and kinematic parameters (VCL, VSL, VAP, BCF, and ALH) compared to the TEY control (p < 0.05). SEM confirmed improved plasma membrane smoothness and reduced structural abnormalities in sperm cryopreserved with TNL, suggesting superior protection against freeze–thaw-induced damage. Moreover, TNL formulations showed reduced sperm DNA fragmentation and better acrosomal preservation, indicating effective stabilization of the lipid bilayer and mitochondrial membranes.

The use of TNL extenders eliminates the dependence on animal-origin materials such as egg yolk, enhancing biosafety and consistency in AI programs. The locally developed NL formulation supports national self-reliance and biosustainability by utilizing indigenous soy lecithin, reducing the need for imported extenders. These findings have broad applicability not only for cattle breeding centers in Indonesia but also for other domestic and wildlife species where semen cryopreservation is crucial for genetic conservation.

This work integrates nanotechnology with reproductive biotechnology, combining particle characterization (size, zeta potential, and morphology) with functional semen evaluation (motility, viability, membrane integrity, acrosome, and DNA quality). It represents the first locally developed, cost-effective NL-based semen extender validated for bull semen cryopreservation in Indonesia. The study provides mechanistic insights into how NLs fuse with sperm membranes, stabilize lipid bilayers, and reduce cryo-induced injury.

The present investigation focused exclusively on *in vitro* sperm quality assessments without *in vivo* fertility trials. Thus, while TNL extenders showed superior physicochemical and cryoprotective performance under laboratory conditions, their actual conception and pregnancy rates in field AI programs remain to be validated. Long-term stability and post-storage functional performance of frozen semen were also not examined beyond the initial evaluation period.

Future studies should include *in vivo* fertility assessments in breeding herds, extended storage trials under routine AI center conditions, and cross-species applications to evaluate the universality of the TNL extender in different livestock and wildlife species. Integration of antioxidant or bioactive nano-additives could further enhance cryostability and sperm function recovery post-thaw. Large-scale field validation will be crucial to establish TNL as a standard biosafe cryoprotectant platform for commercial AI centers.

The TNL extender effectively maintained sperm structural and functional integrity during cryopreservation, outperforming traditional egg-yolk-based extenders. The findings highlight the potential of NLs as multifunctional, biocompatible cryoprotectants capable of replacing animal-derived materials. This innovation supports Indonesia’s move toward sustainable, biosafe, and self-sufficient reproductive biotechnology, contributing to national goals in livestock productivity, genetic conservation, and One Health-aligned (SDG 2: Zero Hunger, SDG 12: Responsible Production and Consumption, and SDG 15: Life on Land).

## AUTHORS’ CONTRIBUTIONS

MG, RIA, EMK, and SS: Conceived the study design, performed the fieldwork, conducted the literature search and drafted the manuscript. MG, RIA, HI, and SS: Conducted data interpretation, performed the statistical analysis, and edited the manuscript. IA, NWKK, MAS, EMK, HI, and SS: Reviewed the manuscript and supervised the study. All authors have read and approved the final version of the manuscript.
